# Depletion of Acetyl‐CoA Carboxylase 1 Facilitates Epithelial‐Mesenchymal Transition in Prostate Cancer Cells by Activating the MAPK/ERK Pathway

**DOI:** 10.1002/mco2.70126

**Published:** 2025-03-10

**Authors:** Jiarun Lai, Shaoyou Liu, Yupeng Chen, Jian Chen, Jinchuang Li, Zhenguo Liang, Xinyue Mei, Yuanfa Feng, Zhaodong Han, Funeng Jiang, Shengbang Yang, Yongding Wu, Huijing Tan, Junchen Liu, Huichan He, Weide Zhong

**Affiliations:** ^1^ Guangdong Provincial Key Laboratory of Urology The First Affiliated Hospital of Guangzhou Medical University Guangzhou China; ^2^ Guangdong Key Laboratory of Clinical Molecular Medicine and Diagnostics The Second Affiliated Hospital School of Medicine South China University of Technology Guangzhou China; ^3^ Guangzhou National Laboratory Guangzhou Guangdong Province China; ^4^ Department of Integrative Biology and Pharmacology McGovern Medical School University of Texas Health Science Center Houston Texas USA; ^5^ Macau Institute for Applied Research in Medicine and Health Macau University of Science and Technology Macau China

**Keywords:** ACACA, EMT, fatty acid biosynthesis, MAPK, metastasis, prostate cancer

## Abstract

Hyperactivation of fatty acid biosynthesis holds promise as a targeted therapeutic strategy in prostate cancer (PCa). However, inhibiting these enzymes could potentially promote metastatic progression in various other cancers. Herein, we found that depletion of acetyl‐CoA carboxylase 1 (encoded by ACACA), the enzyme responsible for the first and rate‐limiting step of de novo fatty acid biosynthesis, facilitated epithelial‐mesenchymal transition (EMT) and migration of PCa cells. This finding was validated in vitro through cell migration assays and in vivo using a metastatic model established by tail vein injection of ACACA‐depleted cells into BALB/c nude mice. Additionally, depletion of ACACA activated the mitogen‐activated protein kinase (MAPK)/extracellular signal‐regulated protein kinases (ERK) pathway. Inhibition of the MAPK/ERK signaling pathway reduced EMT and migration in ACACA‐depleted cells. Our study is the first to indicate targeting ACACA induces an “unexpected” escape program through activation of the MAPK/ERK signaling pathway in PCa, ultimately leading to EMT and metastasis. Therefore, we strongly recommend that the potential adverse effects of targeting ACACA or its derived therapeutic agents must be given extreme attention, especially in MAPK‐related cancers.

## Graphical abstract

Depletion of ACACA in prostate cancer cells inhibits the PI3K‐AKT pathway, leading to negative feedback activation of the MAPK pathway. This activation increases expression of EMT transcription factors, particularly SLUG, resulting in upregulation of mesenchymal biomarkers and down‐regulation of epithelial biomarkers. Consequently, cancer cells exhibit morphological changes and enhanced motility, promoting metastasis. These findings highlight the role of ACACA depletion in driving EMT and metastasis via MAPK pathway activation.

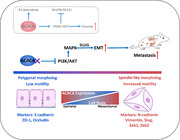

## 1 Introduction

Hyperactivation of de novo fatty acid synthesis has been recognized as a significant metabolic trait in prostate cancer (PCa) [1]. Multiple positron emission tomography/computed tomography (PET/CT)‐based clinical studies indicated that the sensitivity of ^11^C‐acetate (participating in cell membrane lipid synthesis) in detecting PCa is higher than that of the conventional tracer ^18^F‐FDG. This further reveals the significance of fatty acid biosynthesis in PCa [2].

Acetyl‐CoA carboxylase 1 (ACC1, encoded by the ACACA gene) is the first and rate‐limiting enzyme that controls fatty acid biosynthesis activity. Although targeting ACACA inhibits tumor growth and increases apoptosis, it also induces different cell states in cancer cells by activating survival pathways [3, 4]. These findings are significant for the tumor escape observed in clinical trials focused on targeting fatty acid metabolism. Therefore, it is crucial to explore the mechanisms underlying tumor escape.

One potential mechanism underlying escape involves initiation of invasion and metastasis patterns by cancer cells when influenced by various internal or external factors [5]. Cancer cells can transiently or stably undergo epithelial‐mesenchymal transition (EMT). This cellular process involves epithelial cells acquiring mesenchymal phenotypes and behaviors following the downregulation of epithelial characteristics. Expressing an activated set of pleiotropic transcription factors, such as SLUG, ZEB1, and ZEB2, results in alterations in cell biology characteristics, including loss of adhesion junctions, associated morphological transition from polygonal epithelial cells to spindle‐shaped fibroblasts, expression of matrix‐degrading enzymes, and increased motility [6, 7]. We discovered that when the rate‐limiting enzyme in fatty acid synthesis is inhibited, PCa cells tend to undergo EMT and develop evasion strategies.

Further exploration of signaling pathways reveals that the MAPK pathway plays a significant role in lipid metabolism and cancer progression. This occurs via extracellular signal‐regulated kinase (ERK) activity, which phosphorylates key proteins involved in the cell migration machinery [8, 9]. ERK regulates cell migration through lamellipodia formation, actomyosin contractility, and focal adhesion turnover [10]. Inhibitors of mitogen‐activated extracellular signal‐regulated kinase (MEK) and its downstream target ERK, as well as their mutants, have the potential to restrict migration of various cell types [11, 12, 13].

In this study, our result demonstrated that targeting de novo fatty acid biosynthesis by depletion of ACACA triggers EMT in PCa cells, which further promotes cell migration and metastasis. Meanwhile, MAPK/ERK signaling pathway is activated under the above interventions. Significantly, inhibition of MAPK/ERK signaling attenuates EMT and migration of ACACA‐depleted PCa cells. Thus, we suggest that targeting ACACA leads to EMT and metastasis by activating the MAPK/ERK signaling pathway.

## 2 Results

### 2.1 Depletion of ACACA Increases the Metastatic Potential of PCa Cells

**FIGURE 1:**
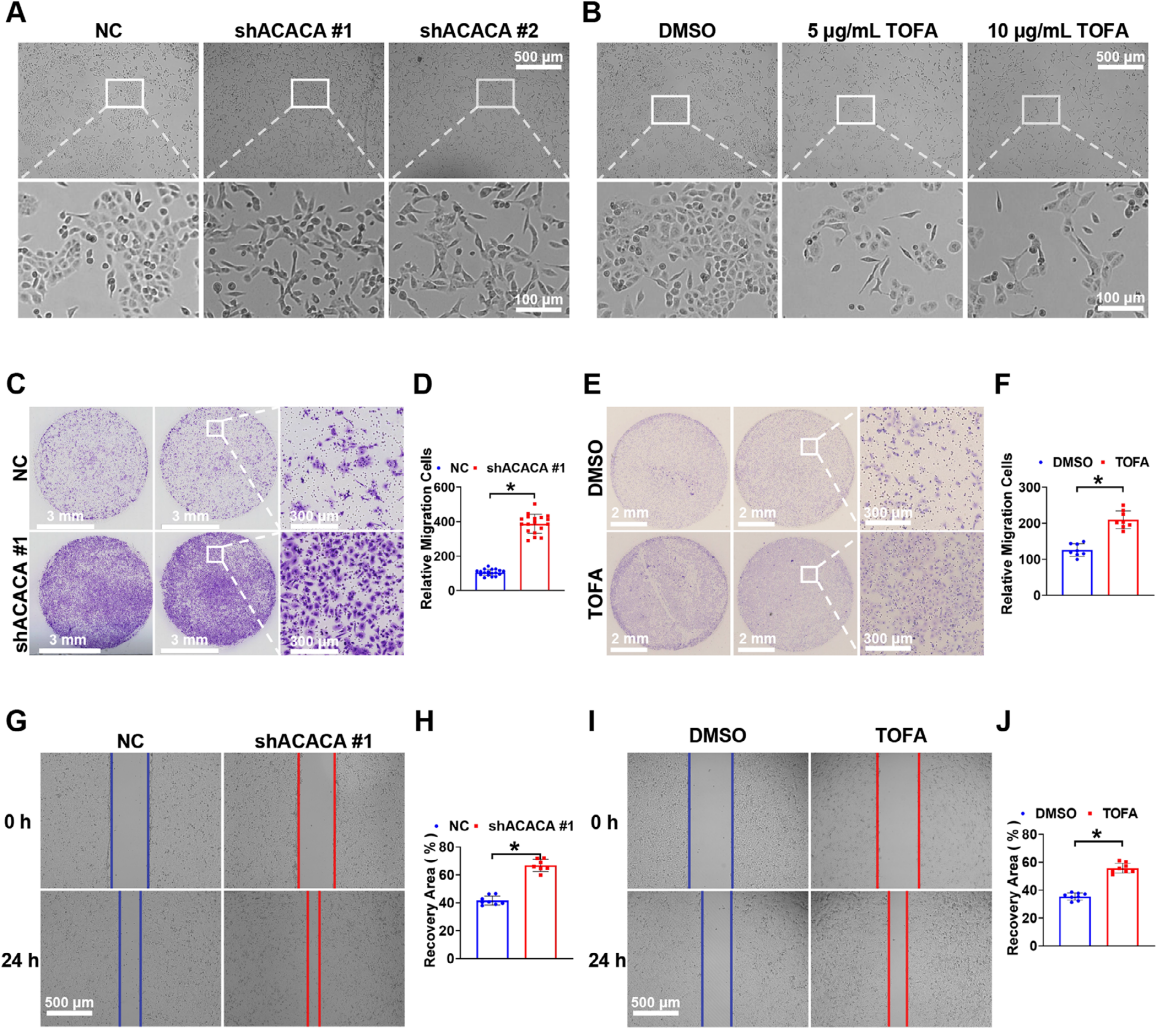
Depletion of ACACA increases the metastatic potential of PCa cells. (A) Representative images of cell morphology in ACACA‐depleted DU145 cells. (B) Representative images of cell morphology 3 days after TOFA (ACACA inhibitor) treatment at specified concentrations. (C and D) Assessment of the migration potential of ACACA‐depleted DU145 cells using Transwell assay. (E and F) Assessment of the migration potential of DU145 cells treated with TOFA using Transwell assay. (G and H) Assessment of the migration potential of ACACA‐depleted DU145 cells using wound healing assay. (I and J) Assessment of the migration potential of DU145 cells treated with TOFA using wound healing assay. Quantification of all the relative data using ImageJ. *p < 0.05.

To determine whether ACACA contributes to the metastatic potential of PCa cells, shRNA (with two different sequences) or TOFA was used to deplete ACACA in DU145 cells. Depletion of ACACA using shRNA resulted in the loss of their cell–cell junctions and made them more motile (Figure 1A). Similarly, cells treated with TOFA at concentrations of 0, 5, or 10 µg/mL for 72 h exhibited the same morphological changes (Figure 1B). Transwell and wound‐healing assays demonstrated that the migration potential was enhanced in the ACACA‐depleted DU145 cells (Figure 1C–J). The results using another shRNA sequence for ACACA depletion are shown in Figure S1.

### 2.2 ACACA Expression Is Negatively Correlated With EMT Signature in PCa

**FIGURE 2:**
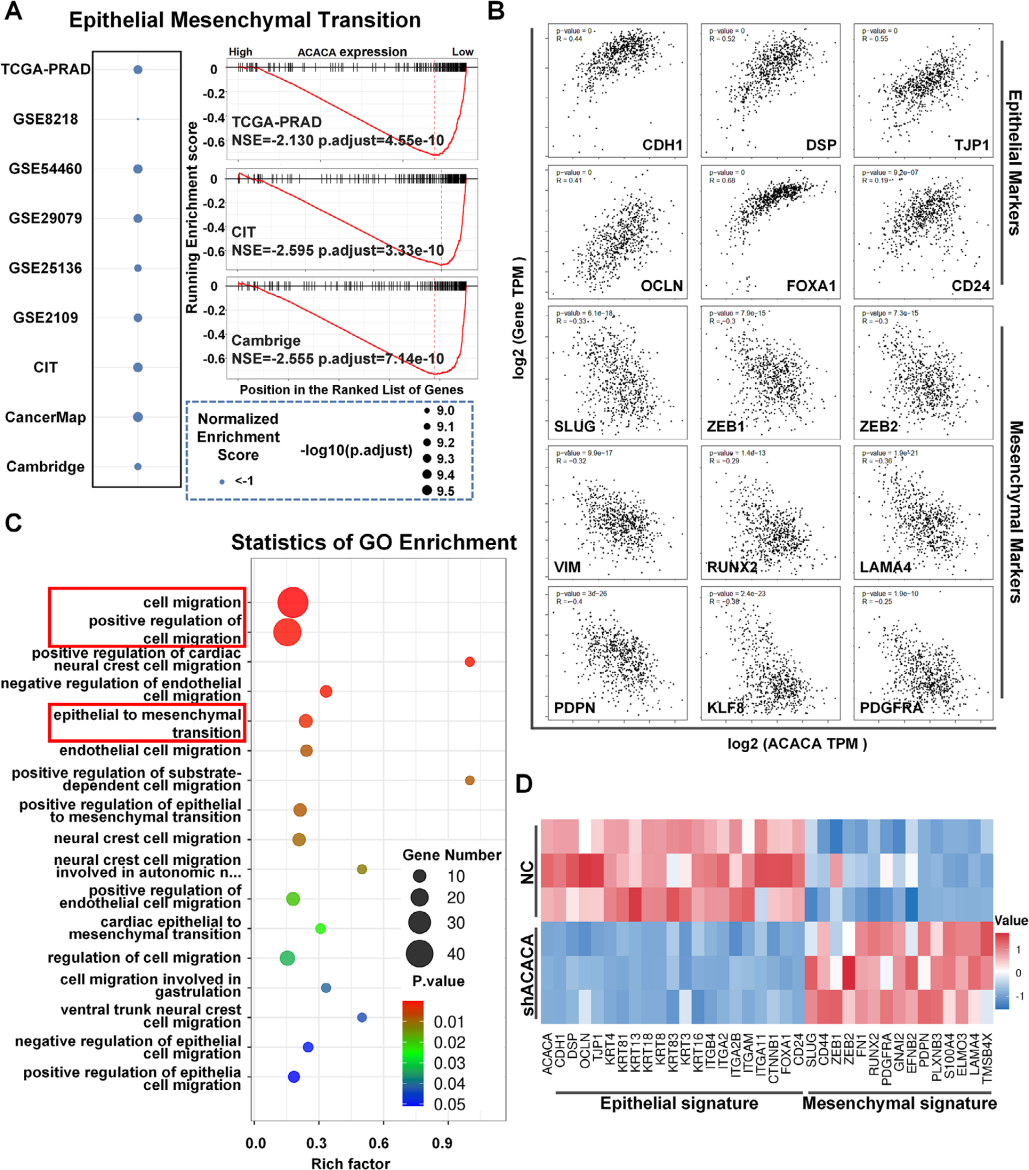
ACACA expression is negatively correlated with EMT signature in PCa. (A) Bubble plot indicates the indicated pathways related to ACACA in nine public datasets. GSEA analysis of indicated ACACA expression and epithelial‐mesenchymal transition (EMT) signaling pathways in specified datasets (The Cancer Genome Atlas [TCGA], Carte d'Identité des Tumeurs [CIT] and Cambridge), with adjusted *p* < 0.05. (B) Correlation analysis between ACACA and EMT‐related markers (CDH1, DSP, TJP1, OCLN, FOXA1, CD24, SLUG, ZEB1, ZEB2, VIM, RUNX2, LAMA4, PDPN, KLF8, and PDGFRA) expression in the TCGA dataset. (C) Pathways significantly enriched in ACACA‐depleted DU145 cells. (D) Heatmap of EMT biomarker‐related gene expression in ACACA‐depleted DU145 cells.

To assess the relationship between ACACA expression and the EMT signaling pathway, we used nine clinical datasets (TCGA, GSE8218, GSE54460, GSE29079, GSE25136, GSE2109, CIT, CancerMap, and Cambridge) for GSEA (Figure 2A and Figure S2). ACACA expression is negatively correlated with EMT signature in PCa. Additionally, we analyzed the significant EMT genes related to ACACA expression. ACACA expression exhibited a positive correlation with epithelial markers, such as CDH1, DSP, TJP1, OCLN, FOXA1, and CD24. In contrast, it was negatively correlated with the expression of mesenchymal cell markers, including SLUG, ZEB1, ZEB2, VIM, RUNX2, LAMA4, PDPN, KLF8, and PDGFRA (Figure 2B). To confirm these findings, RNA‐seq was performed to analyze the alterations in ACACA‐depleted DU145 cells. Bioinformatics analysis indicated that the signaling pathways involved in EMT and cell migration were significantly enriched (Figure 2C). Furthermore, a heatmap demonstrated corresponding alterations in the mRNA expression levels of the representative EMT biomarkers (Figure 2D).

### 2.3 Depletion of ACACA Triggers EMT in PCa Cells

**FIGURE 3:**
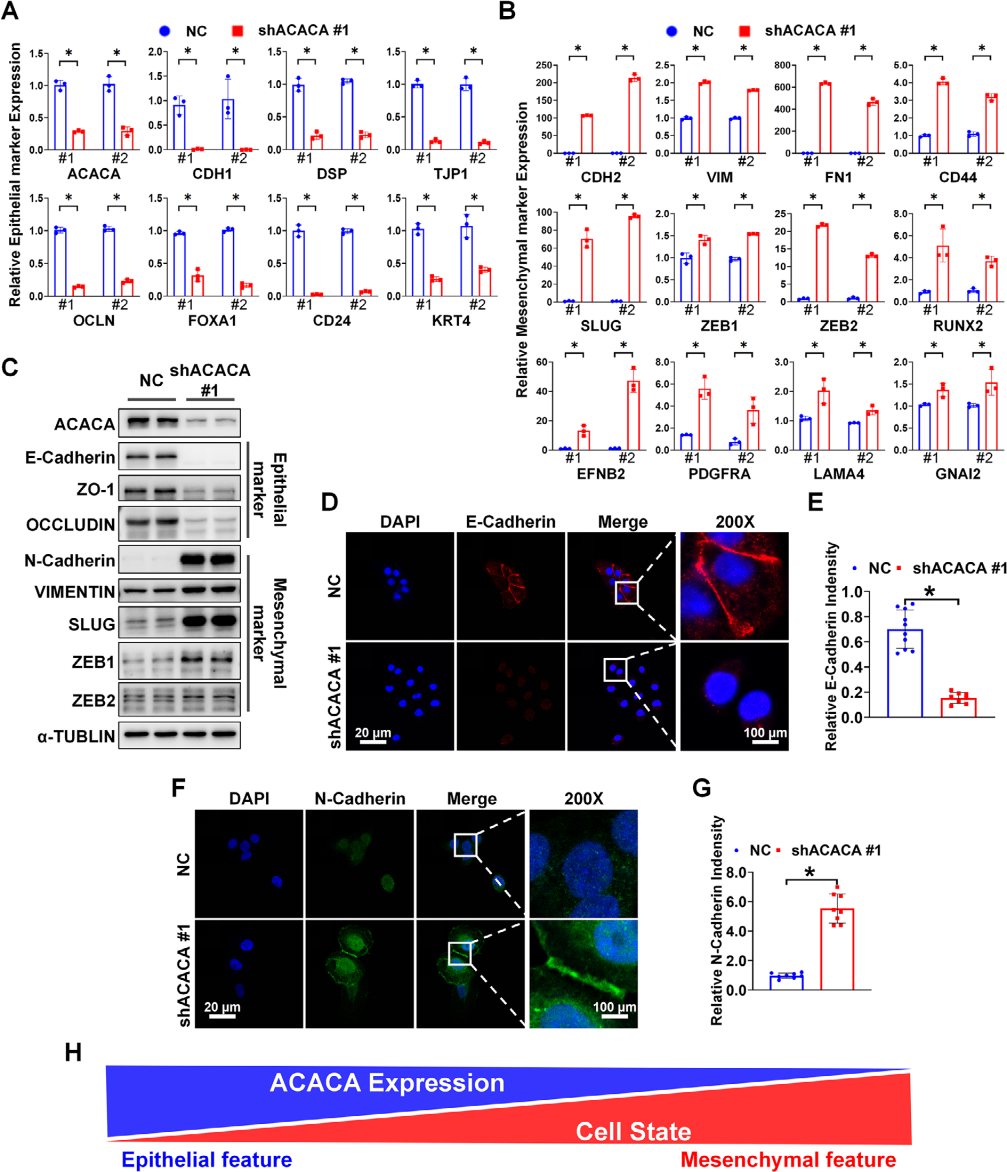
Depletion of ACACA triggers EMT in PCa cells. (A and B) Quantification of qPCR analysis results of EMT biomarker‐related gene expression in ACACA‐depleted DU145 cells, using two primers (#1, primer 1; #2, primer 2). (C) Western blot analysis of the specified protein levels in ACACA‐depleted DU145 cells. (D–G) Immunofluorescence analysis of E‐cadherin and N‐cadherin, respectively, in ACACA‐depleted DU145 cells using confocal microscopy. Quantification of the relative fluorescence intensity using ImageJ. (H) EMT mechanisms regulated by ACACA expression in DU145 cells. *p < 0.05.

qPCR analysis was performed to assess whether depletion of ACACA facilitates EMT in DU145 cells (Figure 3A,B). The protein expression of major epithelial markers (E‐cadherin, ZO‐1, and OCCLUDIN) was downregulated, while mesenchymal markers (N‐cadherin, VIMENTIN, SLUG, ZEB1, and ZEB2) were upregulated (Figure 3C). These results were observed using one of the shRNA sequences for ACACA depletion. The results using another shRNA sequence for ACACA depletion are shown in Figure S3. Subsequently, immunofluorescence staining demonstrated that ACACA‐depleted cells lost intercellular adhesion, and E‐cadherin expression was downregulated (Figure 3D,E). In contrast, N‐cadherin expression was upregulated in the ACACA‐depleted cells (Figure 3F,G). These findings demonstrate that EMT is regulated by ACACA expression in PCa (Figure 3H).

### 2.4 Depletion of ACACA Facilitates the Development of Metastatic PCa

**FIGURE 4:**
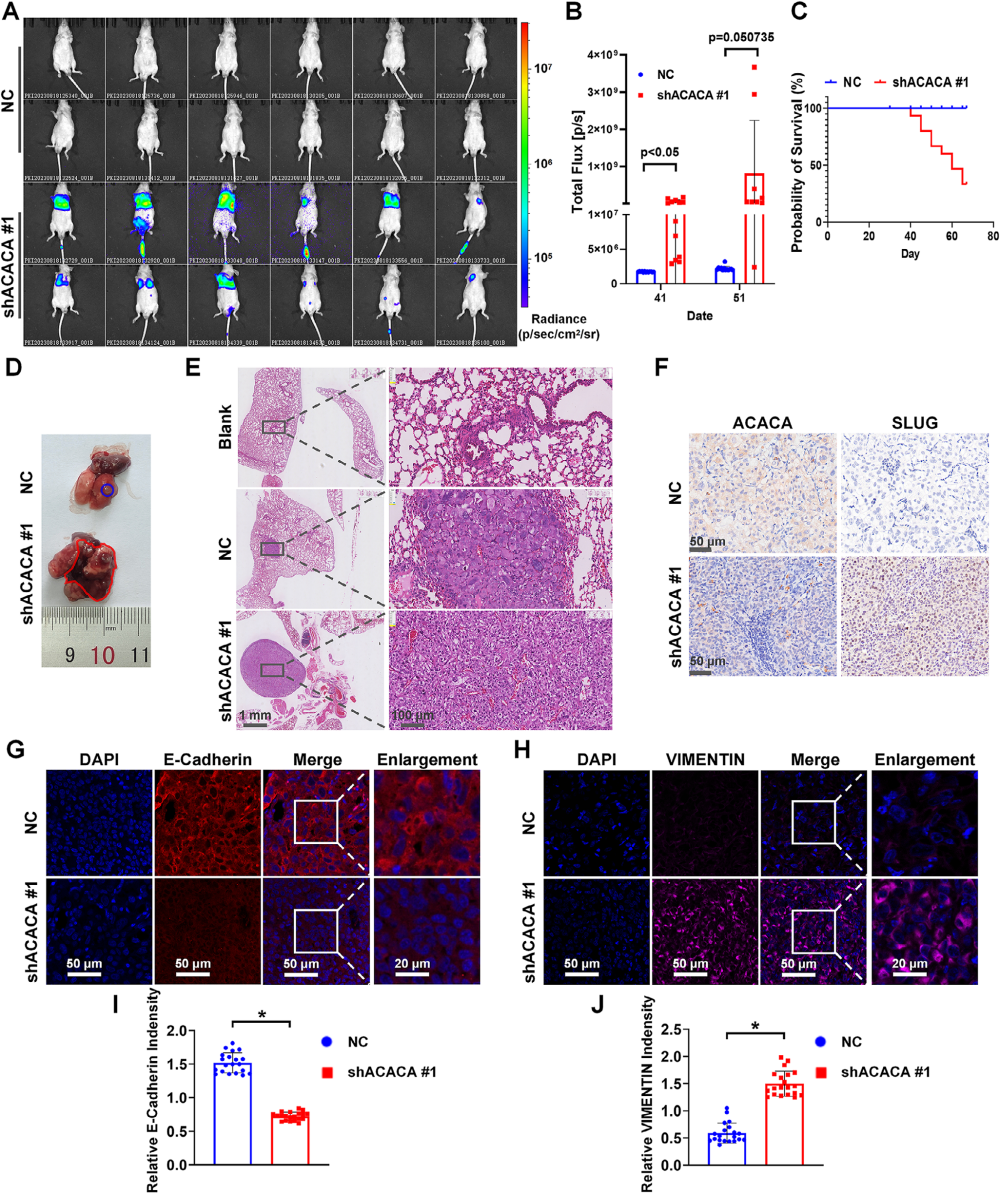
Depletion of ACACA facilitates the development of metastatic PCa. (A) Indicated luciferase‐labeled DU145 cells were injected to BALB/c nude mice via the tail vein, and live images were taken after 41 days. (B) Bioluminescence on days 41 and 51. (C) Cumulative survival analysis of the model from (A). (D and E) Representative images of the gross anatomy and hematoxylin–eosin‐stained mouse lung tissues, respectively. Blank represents the absence of cell injection into the model. (F) Representative images of the immunohistochemistry showing ACACA and SLUG staining in two groups of lung metastases. (G–J) Immunofluorescence analysis of E‐cadherin and VIMENTIN, respectively, using confocal microscopy. Quantification of the relative fluorescence intensity using ImageJ. *p < 0.05.

To assess the effect of ACACA depletion on tumor metastasis in vivo, we used luciferase‐coupled shRNA to knock down the ACACA in DU145 cells. These cells were injected into BALB/c nude mice via the tail vein. Bioluminescence images showed that ACACA‐depleted models developed more metastatic tumors than control cells on day 41 (Figure 4A,B) and day 51 (Figure S4). Mice injected with ACACA‐depleted cells began dying after 41 days. By day 66, more than 50% of the mice had died, whereas none in the control group died (Figure 4C). Anatomical and H&E staining images of the mouse lungs were presented to visually demonstrate metastatic formation (Figure 4D,E). Subsequently, immunohistochemistry (IHC) staining showed elevated SLUG expression in ACACA‐depleted cell‐injected mice (Figure 4F). Immunofluorescence staining showed downregulation of E‐cadherin and upregulation of VIMENTIN in ACACA‐depleted cell‐injected mice (Figure 4G–J). These results demonstrate that ACACA depletion facilitates the development of metastatic PCa in vivo.

### 2.5 Depletion of ACACA Activates MAPK/ERK Pathway in PCa Cells

**FIGURE 5:**
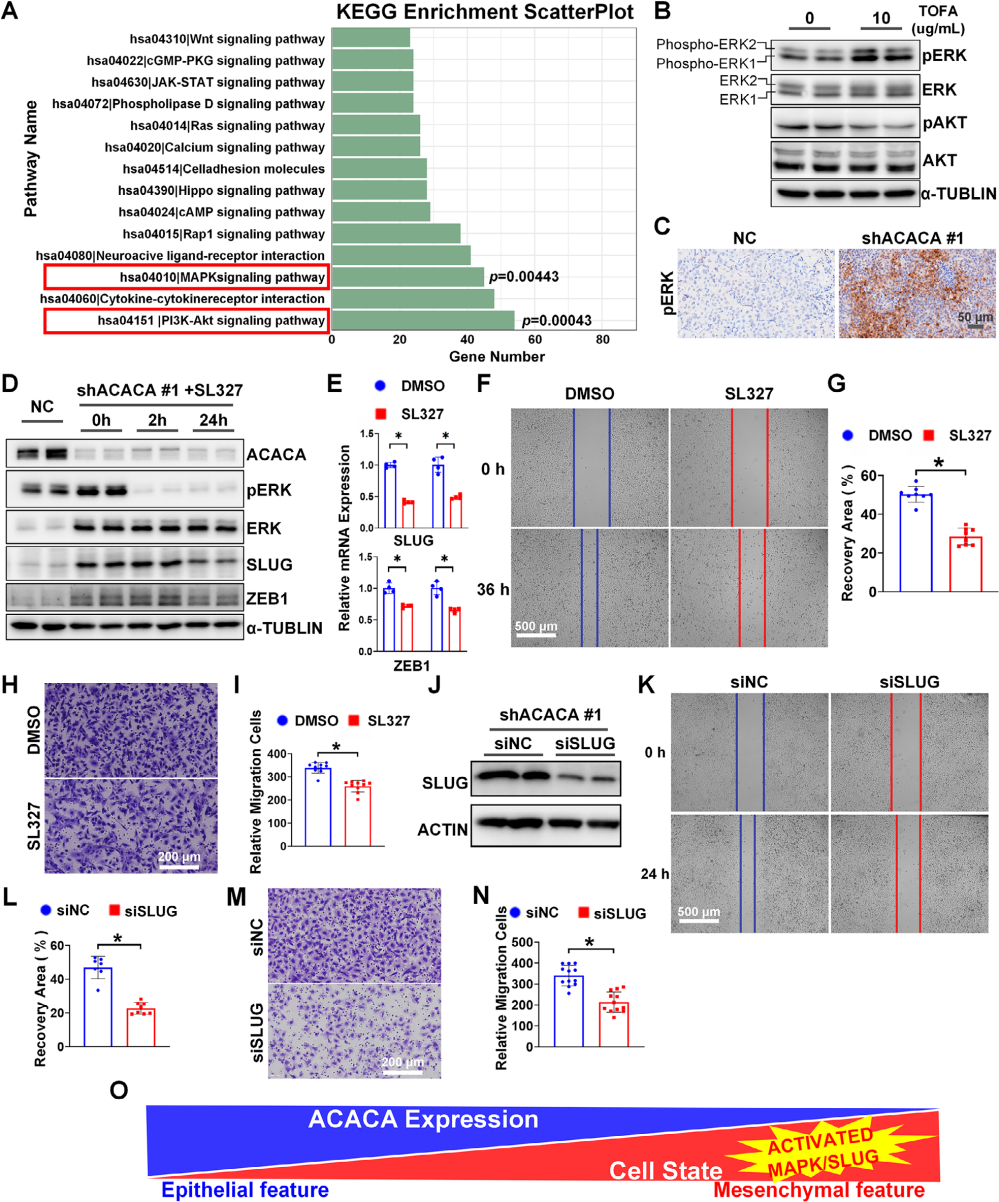
Inhibition of MAPK/SLUG signaling attenuates EMT and migration of ACACA‐depleted PCa cells. (A) Pathways significantly enriched in ACACA‐depleted DU145 cells. (B) Western blot analysis of the specified protein levels in ACACA inhibitor (TOFA, 48 h)‐treated DU145 cells. (C) Representative images of the immunohistochemistry showing pERK staining in two groups of lung metastases in mouse model. (D) Western blot analysis of the specified protein levels in ACACA‐depleted DU145 cells following treatment with a mitogen‐activated extracellular signal–regulated kinase (MEK) inhibitor (SL327, 10 µM). (E) Quantification of qPCR analysis results of the specified gene expression in ACACA‐depleted DU145 cells following MEK inhibitor (SL327, 10 µM, 24 h) treatment. Results obtained using two different primers. (F–I) Assessment of the migration potential of ACACA‐depleted DU145 cells following treatment with MEK inhibitors (SL327, 10 µM; SCH772984, 1 µM) using wound healing and Transwell assays. (J) Western blot analysis of SLUG levels in ACACA‐depleted DU145 cells following SLUG silencing. (K–N) Assessment of the migration potential of ACACA‐depleted DU145 cells following SLUG silencing using Wound healing and Transwell assays. Quantification of the indicated results using ImageJ. (O) Mechanisms of EMT facilitated by ACACA depletion via MAPK/ERK pathway activation in prostate cancer. *p < 0.05.

RNA sequencing of ACACA‐depleted DU145 cells revealed enrichment in KEGG pathways, including the PI3K‐AKT and MAPK pathways (Figure 5A). Similar results were observed in another prostate cancer cell line, PC3, as shown in Figure S5A. Western blot analysis showed that ACACA‐depleted PCa cells (DU145 with shRNA knockdown and TOFA‐treated cells from DU145, PC3, C42, and 22RV1) exhibited higher levels of phosphorylated ERK compared to the control group (Figure 5B, Figure S5B–D). IHC analysis of lung metastases in the mouse model showed increased levels of phosphorylated ERK (Figure 5C).

### 2.6 Inhibition of MAPK/SLUG Signaling Attenuates EMT and Migration of ACACA‐Depleted PCa Cells

The protein (Figure 3C and Figure S3C) and mRNA (Figure 3B and Figure S3B) expression levels of ZEB1 and SLUG were increased in ACACA‐depleted DU145 cells. These levels significantly decreased when ACACA‐depleted cells were treated with SL327, a selective MEK inhibitor (Figure 5D,E and Figure S6A). Based on the previous studies [14] and our RNA sequencing results, EGF, VEGF, and PDGF may serve as negative feedback signals following SL327 treatment (Figure S6B). Transwell and wound healing assays demonstrated that the migration potential of ACACA‐depleted DU145 cells was reduced after treating with SL327 (Figure 5F–I).

Similarly, the protein and mRNA levels of ZEB1 and SLUG were significantly decreased in ACACA‐depleted DU145 cells treated with SCH772984, a highly selective ERK inhibitor (Figure S7A,B). Wound healing assays showed that the migration potential of ACACA‐depleted DU145 cells was reduced following SCH772984 treatment (Figure S7C,D).

Furthermore, both protein and mRNA levels of ZEB1 and SLUG were downregulated in MAPK1‐silenced ACACA‐depleted DU145 cells, with minimal changes in MAPK3‐silenced cells (Figure S7E–H). Wound healing assays also showed a rescue of migration in MAPK1‐silenced ACACA‐depleted DU145 cells (Figure S7I,J).

Both transcription factors SLUG and ZEB1 exhibited changes after ACACA knockdown or rescue with MAPK inhibitors. As shown in the qPCR results (Figure 3B), ZEB1 increased by only 1.5‐fold after ACACA knockdown, whereas SLUG increased by approximately 90‐fold with primer 2. To confirm whether SLUG is crucial in mediating the effects of ACACA targeting, we conducted a series of validations using siRNA. Notably, SLUG knockdown (Figure 5J) significantly reduced the migration potential of ACACA‐depleted DU145 cells (Figure 5K–N).

In conclusion, these results demonstrate that targeting the MAPK/SLUG signaling pathway effectively attenuates EMT and cell migration in ACACA‐depleted PCa cells (Figure 5O).

## 3 Discussion

Therapies targeting fatty acid biosynthesis are promising for the treatment of various cancers [15, 16, 17, 18, 19, 20]. However, this treatment approach has also been shown to have unexpected effects on cancer metastasis, which is lethal to patients with cancer [21, 22]. Expression of ACACA, the rate‐limiting enzyme of fatty acid biosynthesis, in a PCa tissue microarray has been reported to be significantly higher than in non‐malignant tissue and inhibition of this enzyme inhibits tumor growth and increases apoptosis in PCa [3, 4]. However, the escape pathways following targeted fatty acid therapy in PCa remain unclear. In this study, we present novel findings that depletion of ACACA facilitates EMT through MAPK/ERK pathway activation, thereby facilitating the migration and metastasis of PCa cells (as shown graphical abstract figure).

EMT plays a critical role in the migration of cancer cells and their resistance to therapies, thereby contributing to enhanced metastasis. Our results demonstrated that the inhibition of ACACA expression in PCa cells resulted in the loss of cell adhesion function, an associated morphological transition from polygonal epithelial cells to spindle‐shaped fibroblasts, and enhanced cell motility. According to these findings, EMT activation leads to a series of biological cellular characteristics [6]. Specifically, depletion of ACACA was shown to activate EMT in PCa. Using the GSEA public database, we discovered that low ACACA expression activated the EMT pathway in PCa. Additionally, we discovered that alterations in marker gene expression in ACACA‐depleted PCa cells resulted in the transition from an epithelial state to a mesenchymal state. When we knocked down the expression of SLUG, a key transcription factor involved in EMT, the migration potential of ACACA‐depleted cells was significantly reduced. As anticipated, we confirmed that depletion of ACACA facilitated the development of metastatic PCa in vivo.

In tumor cells, PI3K‐AKT activation induces phosphorylation of proapoptotic family members and inhibits apoptosis, while ERK1/2 signaling promotes cell survival [23]. From the RNA sequencing data analysis, we observed that two pathways closely related to prostate cancer progression, the PI3K‐AKT pathway and the MAPK pathway [10, 24–27], were significantly enriched among the differentially expressed genes following ACACA knockdown (Figure 5A and Figure S5A). Our previous research has reported that ACACA knockdown in prostate cancer cells leads to a significant decrease in de novo fatty acid synthesis, which notably inhibits the PI3K‐AKT pathway, causing the cells to experience pronounced metabolic stress [4]. However, substantial evidence suggests that there is a negative feedback loop between the PI3K/AKT pathway and the MAPK pathway [25, 28–30]. When the PI3K/AKT pathway is inhibited, the suppression of the MAPK signaling pathway is relieved [31, 32], resulting in its activation and further promoting prostate cancer progression. Based on these findings, we further investigated and validated that ACACA knockdown significantly activates the MAPK/ERK pathway.

ERK is primarily encoded by two genes, MAPK1 and MAPK3, which produce the proteins ERK2 and ERK1, respectively. Herein, we demonstrated that inhibiting the upstream molecule MEK, ERK itself, and specifically ERK2 (MAPK1) all trigger the EMT program, leading to ACACA‐depleted prostate cancer cell migration. Our results showed that SLUG expression was markedly upregulated following ACACA knockdown, with the most significant increase observed among the transcription factors. Furthermore, this upregulation was reversed by treatment with a MAPK inhibitor. To further confirm the importance of SLUG in this pathway, we conducted siRNA‐mediated knockdown of SLUG and found that this significantly reduced the migration potential of ACACA‐depleted DU145 cells. Together, these findings highlight the critical role of the MAPK/ERK pathway in triggering the EMT program and driving migration in ACACA‐depleted PCa cells.

Considering the above, we propose that ACACA knockdown causes prostate cancer cells to endure long‐term metabolic stress. The adaptive capacity of these cells drives them to activate survival‐related signaling pathways, leading to EMT changes and metastasis. Due to the potential of EMT to foster escape to ACACA‐targeted therapies, we highly advise that careful attention be paid to the adverse effects mentioned above, as well as to the therapeutic agents derived from them, particularly in the case of MAPK‐related prostate cancers.

Exploring the metabolic and lipid environments in PCa, and identifying nutrients or therapeutic agents that could restrict the growth of metastatic cancer cells, represent a promising avenue for therapeutic intervention [33, 34, 35]. However, determining the specific type of prostate cancer metastasis that exhibits similar characteristics to those found in this study will require further investigation (Figure S8A). Additionally, while prior reports have suggested that the activation of the MAPK signaling pathway in PCa cells may be influenced by certain fatty acid metabolism substances [36, 37], the exact mechanisms through which these substances affect MAPK signaling still require in‐depth research (Figure S8B).

## 4 Materials and Methods

### 4.1 Cell Culture

Human PCa cell lines, including DU145, PC3, 22RV1, and C42, and human embryonic kidney (HEK) 293T cells were purchased from the American Type Culture Collection. All cell lines used in this study have undergone authentication by short tandem repeat (STR) analysis to confirm their identity and tested mycoplasma free. The cells were cultured in Dulbecco's modified Eagle's medium (DMEM) (Gibco, 10270‐106) supplemented with 10% fetal bovine serum (FBS) (Gibco, 10270‐106) and 1% penicillin‐streptomycin (Gibco, 15140122) in a CO_2_ incubator (5 %) at 37°C. For the cell signaling assay, the ACACA allosteric inhibitor 5‐tetradecyloxy‐2‐furoic acid (TOFA) (Abcam, ab141578), MEK inhibitor SL327 (Sellcek, S1066), and ERK inhibitor SCH772984 (Sellcek, S7101) were added to the medium at specified concentrations.

### 4.2 Cell Line Construction

Short hairpin RNA (shRNA) plasmids were purchased from Kelei Biological Technology Co., Ltd. Plasmid and virus packaging vectors psPAX2, pMD2.G, and TransfectTurbo (Thermo Fisher Scientific, 01300669) were used for lentiviral packaging in HEK293T cell lines. Two days after lentiviral infection, the cells were selected by growing in a medium supplemented with puromycin (2 µg/mL). Subsequently, single clones of transduced cells were selected for verification and analysis.

siRNAs were purchased from Guangzhou IGE Biotechnology Co. Ltd. and was mixed with the transfection reagent siRNAmate (GenePharm, G04002) following the manufacturer's instructions, and the cells were incubated for 72 h. The shRNA and siRNA sequences used are listed in Table S1.

### 4.3 Bioinformatics Analysis

RNA sequencing data for 495 patients with PCa were retrieved from The Cancer Genome Atlas (TCGA) Prostate Adenocarcinoma (TCGA‐PRAD) database via http://xenabrowser. net/datapages/. This was processed using the R package GDCRNAtools. The CIT (E‐MTAB‐6128) dataset comprising mRNA counts and clinical data of 141 patients with PCa was obtained from ArrayExpress (https://www.ebi.ac.uk/arrayexpress/). mRNA expression data from seven public datasets (GSE8218, GSE54460, GSE29079, GSE25136, GSE2109, CancerMap, and Cambridge) were acquired from Gene Expression Omnibus (GEO; https://www.ncbi.nlm.nih.gov/geo/). We used Pearson's correlation analysis to estimate the correlation coefficient between ACACA and significant EMT gene expression in TCGA database.

To classify patients from the TCGA‐PRAD into high and low groups based on their fatty acid biosynthesis activity, consensus clustering analysis was conducted based on the expression matrix of fatty acid biosynthesis‐related genes (FASRGs) with the R package “ConsensusClusterPlus.” Eighty FASRGs were downloaded for the Gene Ontology term “fatty acid biosynthetic process” (GO:0006633).

Gene set enrichment analysis (GSEA), a differential expression analysis, was initially performed between the high‐ and low‐expression subgroups based on the median expression levels of the specified genes in each dataset. Genes were ranked based on reducing Log2Foldchange levels. The ordered gene list was further subjected to GSEA using the R package “clusterProfilter.” Pathways with an adjusted *p* < 0.05 and a normalized enrichment score (NES) >1 were considered activated, whereas pathways with NES < −1 were considered suppressed. NES and false discovery rate values are shown in the figures.

### 4.4 Western Blotting

Cell lysates were prepared by homogenization in radioimmunoprecipitation assay (RIPA) buffer containing phenylmethylsulfonyl fluoride (PMSF) protease inhibitor (100×) (Beyotime, #ST506) and phosphatase inhibitor (50×) (BoCai Biology, #R0127). The lysates were mixed with SDS loading buffer (5×) (Biosharp, #BL502A) and boiled for 10 min to generate samples. The proteins were separated using SDS‐PAGE and blotted onto polyvinylidene difluoride membranes following the protocol detailed in our previous study [3]. The membranes were incubated with primary antibodies overnight at 4°C and secondary antibodies for 1 h at room temperature (RT). Membrane signals were detected using a ChemiDoc MP Imaging System (BIO‐RAD, Hercules, CA, USA, #12003154). The antibodies used are listed in Table S2.

### 4.5 RNA Expression

NucleoZol (MN, #740404.200) was used to extract RNA from the cells following the manufacturer's instructions. The concentration and quality of RNA were measured using a spectrophotometer. cDNA was generated using HiScript III RT SuperMix after removing residual genomic DNA using qPCR (+gDNA wiper) (Vazyme, #R323). cDNA, primers, and 2× ChamQ Universal SYBR qPCR Master Mix (Vazyme, #Q711) were mixed and added to a qPCR 96‐well plate containing cDNAs. They were amplified using Applied Biosystem QuantStudio 1 system (Thermo Fisher Scientific, Waltham, MA, USA, #A40427), and the real‐time fluorescence intensity was detected. Relative mRNA levels were normalized to those of beta actin (ACTB). Primer sequences are listed in Table S3.

### 4.6 Histology

Lung tissues from nude mice were fixed in paraformaldehyde (4 %) for 16 h, dehydrated, dewaxed, and embedded in paraffin. Tissues were sectioned (4 µm), mounted on glass slides, and dried in an oven at 60°C for 2 h. Hematoxylin–eosin (H&E) and immunohistochemical (IHC) staining was performed as previously described [38]. For fluorescent immunocytochemistry (ICC) staining, the cells were fixed in paraformaldehyde (4%) following incubation in confocal plates and stained following our previous protocol [3]. The primary antibodies used for ICC were anti‐E‐cadherin (Proteintech, #20874‐1‐AP) (1:400), anti‐N‐cadherin (CST, #13116) (1:200), and anti‐VIMENTIN (CST, #5741) (1:200) and primary antibodies for IHC were anti‐ACACA (Proteintech, #67373‐1), anti‐SLUG (CST, #9585), and anti‐pERK (CST, #4370), with concentrations determined according to the product datasheets.

### 4.7 Wound‐Healing Assay

Following digestion and resuspension, 1.5 × 10^6^ cells/well were seeded in six‐well plates to form a confluent monolayer the following day. A 200 µL tip was used to scratch a cell‐free gap. Subsequently, the cells were washed with PBS, and a new serum‐free medium was added. Images were taken at specified intervals and analyzed using ImageJ software version V1.8.0.112 (National Institute of Health, Bethesda, MD, USA) to quantify the relative area of cell migration.

### 4.8 Transwell Assay

The cells were digested, resuspended, and counted. To the lower chamber of the Transwell plate, DMEM (600 µL) containing FBS (10 %) was added, whereas the upper chamber was filled with serum‐free DMEM (200 µL) containing 3 × 10^4^ cells. Following a 24‐h incubation, the membranes in the upper chamber were fixed with paraformaldehyde (4%) for 15 min and stained with gentian violet (0.1%) for 20 min. The cells from the upper side of the membrane were removed using a cotton swab. Following washing and drying, the chamber membrane was removed, placed on a glass slide, and sealed with neutral balsam. The cell count was analyzed after scanning the slides.

### 4.9 Mouse Metastasis Model

DU145‐luciferase (DU145‐Luc) cells were generated using the lentivirus system outlined in the cell line construction method. Five‐week‐old BALB/c nude mice were divided into experimental groups, and 1.5 × 10^6^ DU145‐Luc cells were diluted in sterile PBS (100 µL) and administered through the tail vein using an insulin syringe. Bioluminescence was measured 18 min after IP luciferin administration (Yeasen, #40901ES) using an in vivo imaging system (IVIS), following the manufacturer's instructions. The mice were housed in accordance with the principles and procedures of the Guide for the Care and Use of Laboratory Animals, and the experiments were approved by the South China University of Technology.

### 4.10 RNA‐seq

TRIzol reagent (Invitrogen, CA, USA, #50175111) was used to extract RNA from the cells. The amount and purity of the RNA were quantified using a NanoDrop ND‐1000 (NanoDrop, Wilmington, DE, USA). The final cDNA library was constructed from cleaved small RNA fragment reverse‐transcribed cDNA. Paired‐end sequencing (PE150) was performed on the Illumina NovaSeq 6000 (LC‐Bio Technology Co., Ltd., Hangzhou, China) following the manufacturer's instructions. The entire RNA‐seq process was performed by the LC‐Bio Technology Co. Ltd. Assessment of sequence quality and bioinformatics analysis of RNA‐seq were performed as outlined in a previous study [3].

### 4.11 Statistical Analysis

All experiments were independently repeated at least thrice. GraphPad Prism 9.0 (GraphPad, San Diego, CA, USA) was used to perform statistical analysis and data visualization. Statistical differences between groups were assessed using an independent two‐tailed *t*‐test. The mean ± standard deviation (SD) was represented as error bars, and *p* < 0.05 indicated statistical significance.

## Author Contributions

Conceptualization: Weide Zhong, Huichan He, Shaoyou Liu, and Jiarun Lai. Methodology: Jiarun Lai, Yupeng Chen, and Jian Chen. Validation: Jiarun Lai, Yupeng Chen, Jian Chen, Jinchuang Li, Zhenguo Liang, Xinyue Mei, Yuanfa Feng, Zhaodong Han, Funeng Jiang, Shengbang Yang, Yongding Wu, and Huijing Tan. Data Curation: Jiarun Lai, Shaoyou Liu, Yupeng Chen, and Jian Chen. Supervision: Weide Zhong, Huichan He, and Shaoyou Liu. Writing – original draft: Jiarun Lai and Shaoyou Liu. Writing – review and editing: Weide Zhong, Huichan He, Shaoyou Liu, and Junchen Liu. Project Administration: Weide Zhong and Shaoyou Liu. Funding Acquisition: Weide Zhong. All authors have read and approved the final manuscript.

## Ethics Statement

All experiments involving animals were conducted according to the ethical policies and procedures approved by the South China University of Technology (Approval no. S‐2023‐078‐01).

## Conflicts of Interest

The authors declare no conflicts of interest.

## Supporting information



Supporting Information

## Data Availability

The datasets used and analyzed during the current study are available from the corresponding author on reasonable request.

## References

[1] L. A. Sena and S. R. Denmeade , “Fatty Acid Synthesis in Prostate Cancer: Vulnerability or Epiphenomenon?,” Cancer Research 81, no. 17 (2021): 4385–4393. 34145040 10.1158/0008-5472.CAN-21-1392 PMC8416800

[2] C. Spick , K. Herrmann , and J. Czernin , “Evaluation of Prostate Cancer With 11C‐Acetate PET/CT,” Journal of Nuclear Medicine 57, no. S3 (2016): 30S–37S. 27694168 10.2967/jnumed.115.169599

[3] H. Zhang , S. Liu , Z. Cai , et al., “Down‐Regulation of ACACA Suppresses the Malignant Progression of Prostate Cancer Through Inhibiting Mitochondrial Potential,” Journal of Cancer 12, no. 1 (2021): 232–243. 33391420 10.7150/jca.49560 PMC7738814

[4] S. Liu , J. Lai , Y. Feng , et al., “Acetyl‐CoA Carboxylase 1 Depletion Suppresses De Novo Fatty Acid Synthesis and Mitochondrial β‐Oxidation in Castration‐Resistant Prostate Cancer Cells,” Journal of Biological Chemistry 299, no. 1 (2023): 102720. 36410440 10.1016/j.jbc.2022.102720 PMC9771725

[5] D. Hanahan and R. A. Weinberg , “Hallmarks of Cancer: The Next Generation,” Cell 144, no. 5 (2011): 646–674. 21376230 10.1016/j.cell.2011.02.013

[6] J. Yang , P. Antin , G. Berx , et al., “Guidelines and Definitions for Research on Epithelial‐Mesenchymal Transition,” Nature Reviews Molecular Cell Biology 21, no. 6 (2020): 341–352. 32300252 10.1038/s41580-020-0237-9 PMC7250738

[7] Y. Huang , W. Hong , and X. Wei , “The Molecular Mechanisms and Therapeutic Strategies of EMT in Tumor Progression and Metastasis,” Journal of Hematology & Oncology 15, no. 1 (2022): 129. 36076302 10.1186/s13045-022-01347-8 PMC9461252

[8] C. Abate‐Shen , “Prostate Cancer Metastasis—Fueled by Fat?,” New England Journal of Medicine 378, no. 17 (2018): 1643–1645. 29694822 10.1056/NEJMcibr1800808 PMC6153443

[9] M. Chen , J. Zhang , K. Sampieri , et al., “An Aberrant SREBP‐Dependent Lipogenic Program Promotes Metastatic Prostate Cancer,” Nature Genetics 50, no. 2 (2018): 206–218. 29335545 10.1038/s41588-017-0027-2 PMC6714980

[10] H. Lavoie , J. Gagnon , and M. Therrien , “ERK Signalling: A Master Regulator of Cell Behaviour, Life and Fate,” Nature Reviews Molecular Cell Biology 21, no. 10 (2020): 607–632. 32576977 10.1038/s41580-020-0255-7

[11] H. Chen , G. Zhu , Y. Li , et al., “Extracellular Signal‐Regulated Kinase Signaling Pathway Regulates Breast Cancer Cell Migration by Maintaining Slug Expression,” Cancer Research 69, no. 24 (2009): 9228–9235. 19920183 10.1158/0008-5472.CAN-09-1950 PMC2795125

[12] C. Choi and D. M. Helfman , “The Ras‐ERK Pathway Modulates Cytoskeleton Organization, Cell Motility and Lung Metastasis Signature Genes in MDA‐MB‐231 LM2,” Oncogene 33, no. 28 (2014): 3668–3676. 23995792 10.1038/onc.2013.341

[13] A. A. Brahmbhatt and R. L. Klemke , “ERK and RhoA Differentially Regulate Pseudopodia Growth and Retraction During Chemotaxis,” Journal of Biological Chemistry 278, no. 15 (2003): 13016–13025. 12571246 10.1074/jbc.M211873200

[14] M. E. Bahar , H. J. Kim , and D. R. Kim , “Targeting the RAS/RAF/MAPK Pathway for Cancer Therapy: From Mechanism to Clinical Studies,” Signal Transduction and Targeted Therapy 8, no. 1 (2023): 455. 38105263 10.1038/s41392-023-01705-z PMC10725898

[15] R. U. Svensson , S. J. Parker , L. J. Eichner , et al., “Inhibition of Acetyl‐CoA Carboxylase Suppresses Fatty Acid Synthesis and Tumor Growth of Non‐Small‐Cell Lung Cancer in Preclinical Models,” Nature Medicine 22, no. 10 (2016): 1108–1119. 10.1038/nm.4181 PMC5053891 27643638

[16] J. S. V. Lally , S. Ghoshal , D. K. DePeralta , et al., “Inhibition of Acetyl‐CoA Carboxylase by Phosphorylation or the Inhibitor ND‐654 Suppresses Lipogenesis and Hepatocellular Carcinoma,” Cell Metabolism 29, no. 1 (2019): 174–182.e5. 30244972 10.1016/j.cmet.2018.08.020 PMC6643297

[17] H. Ito , I. Nakamae , J. Y. Kato , et al., “Stabilization of Fatty Acid Synthesis Enzyme Acetyl‐CoA Carboxylase 1 Suppresses Acute Myeloid Leukemia Development,” Journal of Clinical Investigation 131, no. 12 (2021): e141529. 34128473 10.1172/JCI141529 PMC8203453

[18] G. Falchook , J. Infante , H. T. Arkenau , et al., “First‐In‐Human Study of the Safety, Pharmacokinetics, and Pharmacodynamics of First‐In‐class Fatty Acid Synthase Inhibitor TVB‐2640 Alone and With a Taxane in Advanced Tumors,” EClinicalMedicine 34 (2021): 100797. 33870151 10.1016/j.eclinm.2021.100797 PMC8040281

[19] W. Kelly , A. E. Diaz Duque , J. Michalek , et al., “Phase II Investigation of TVB‐2640 (Denifanstat) With bevacizumab in Patients With First Relapse High‐Grade Astrocytoma,” Clinical Cancer Research 29, no. 13 (2023): 2419–2425. 37093199 10.1158/1078-0432.CCR-22-2807 PMC10320469

[20] Y. Fu , T. Zou , X. Shen , et al., “Lipid Metabolism in Cancer Progression and Therapeutic Strategies,” MedComm 2, no. 1 (2020): 27–59. 34766135 10.1002/mco2.27 PMC8491217

[21] M. Rios Garcia , B. Steinbauer , K. Srivastava , et al., “Acetyl‐CoA Carboxylase 1‐Dependent Protein Acetylation Controls Breast Cancer Metastasis and Recurrence,” Cell metabolism 26, no. 6 (2017): 842–855.e5. 29056512 10.1016/j.cmet.2017.09.018

[22] L. Jiang , L. Xiao , H. Sugiura , et al., “Metabolic Reprogramming During TGFβ1‐Induced Epithelial‐to‐Mesenchymal Transition,” Oncogene 34, no. 30 (2015): 3908–3916. 25284588 10.1038/onc.2014.321 PMC4387121

[23] B. A. Carneiro and W. S. El‐Deiry , “Targeting Apoptosis in Cancer Therapy,” Nature Reviews Clinical Oncology 17, no. 7 (2020): 395–417. 10.1038/s41571-020-0341-y PMC8211386 32203277

[24] E. G. Bluemn , I. M. Coleman , J. M. Lucas , et al., “Androgen Receptor Pathway‐Independent Prostate Cancer Is Sustained Through FGF Signaling,” Cancer Cell 32, no. 4 (2017): 474–489.e6. 29017058 10.1016/j.ccell.2017.09.003 PMC5750052

[25] D. J. Mulholland , N. Kobayashi , M. Ruscetti , et al., “Pten Loss and RAS/MAPK Activation Cooperate to Promote EMT and Metastasis Initiated From Prostate Cancer Stem/Progenitor Cells,” Cancer Research 72, no. 7 (2012): 1878–1889. 22350410 10.1158/0008-5472.CAN-11-3132 PMC3319847

[26] R. Ullah , Q. Yin , A. H. Snell , and L. Wan , “RAF‐MEK‐ERK Pathway in Cancer Evolution and Treatment,” Seminars in Cancer Biology 85 (2022): 123–154. 33992782 10.1016/j.semcancer.2021.05.010

[27] C. W. Kinkade , M. Castillo‐Martin , A. Puzio‐Kuter , et al., “Targeting AKT/mTOR and ERK MAPK Signaling Inhibits Hormone‐Refractory Prostate Cancer in a Preclinical Mouse Model,” Journal of Clinical Investigation 118, no. 9 (2008): 3051–3064. 18725989 10.1172/JCI34764 PMC2518074

[28] Q. Wu , Y. B. Wang , X. W. Che , et al., “Junctional Adhesion Molecule‐Like Protein as a Novel Target for Kaempferol to Ameliorate Lung Adenocarcinoma,” Journal of Integrative Medicine 21, no. 3 (2023): 268–276. 37069006 10.1016/j.joim.2023.03.009

[29] M. Scaltriti , L. Prudkin , et al., “PI3K Inhibition Results in Enhanced HER Signaling and Acquired ERK Dependency in HER2‐Overexpressing Breast Cancer,” Oncogene 30, no. 22 (2011): 2547–2557. 21278786 10.1038/onc.2010.626 PMC3107390

[30] A. Carracedo , L. Ma , J. Teruya‐Feldstein , et al., “Inhibition of mTORC1 Leads to MAPK Pathway Activation Through a PI3K‐Dependent Feedback Loop in Human Cancer,” Journal of Clinical Investigation 118, no. 9 (2008): 3065–3074. 18725988 10.1172/JCI34739 PMC2518073

[31] Q. Li , Z. Li , T. Luo , et al., “Targeting the PI3K/AKT/mTOR and RAF/MEK/ERK Pathways for Cancer Therapy,” Molecular Biomedicine 3 (2022): 47. 36539659 10.1186/s43556-022-00110-2 PMC9768098

[32] M. C. Mendoza , E. E. Er , and J. Blenis , “The Ras‐ERK and PI3K‐mTOR Pathways: Cross‐Talk and Compensation,” Trends in Biochemical Sciences 36, no. 6 (2011): 320–328. 21531565 10.1016/j.tibs.2011.03.006 PMC3112285

[33] L. W. S. Finley , “What Is Cancer Metabolism?,” Cell 186, no. 8 (2023): 1670–1688. 36858045 10.1016/j.cell.2023.01.038 PMC10106389

[34] F. C. E. Vogel , A. B. Chaves‐Filho , and A. Schulze , “Lipids as Mediators of Cancer Progression and Metastasis,” Nature Cancer 5, no. 1 (2024): 16–29. 38273023 10.1038/s43018-023-00702-z

[35] A. Du , Z. Wang , T. Huang , et al., “Fatty Acids in Cancer: Metabolic Functions and Potential Treatment,” MedComm—Oncology 2 (2023): e25.

[36] J. Xu , S. Lamouille , and R. Derynck , “TGF‐Beta‐Induced Epithelial to Mesenchymal Transition,” Cell Research 19, no. 2 (2009): 156–172. 19153598 10.1038/cr.2009.5 PMC4720263

[37] P. Yang , C. Su , X. Luo , et al., “Dietary Oleic Acid‐Induced CD36 Promotes Cervical Cancer Cell Growth and Metastasis via Up‐Regulation Src/ERK Pathway,” Cancer Letters 438 (2018): 76–85. 30213558 10.1016/j.canlet.2018.09.006

[38] J. Liu , G. Chen , Z. Liu , et al., “Aberrant FGFR Tyrosine Kinase Signaling Enhances the Warburg Effect by Reprogramming LDH Isoform Expression and Activity in Prostate Cancer,” Cancer Research 78, no. 16 (2018): 4459–4470. 29891507 10.1158/0008-5472.CAN-17-3226 PMC6095720

