# Supplementary material for: Depletion of Acetyl‐CoA Carboxylase 1 Facilitates Epithelial‐Mesenchymal Transition in Prostate Cancer Cells by Activating the MAPK/ERK Pathway
Source: MedComm (2020). 2025 Mar 10;6(3):e70126. doi: 10.1002/mco2.70126 (PMC11892147; doi:10.1002/mco2.70126)
Supplement: Supplementary file 1 — Supporting Information [file MCO2-6-e70126-s001.pdf]

Depletion of Acetyl-CoA carboxylase 1 Facilitates Epithelial-Mesenchymal Transition in Prostate Cancer Cells by Activating the MAPK/ERK Pathway

Authors

Jiarun Lai[1,#], Shaoyou Liu[1,#,*], Yupeng Chen[1,#], Jian Chen[1,#], Jinchuang Li[2], Zhenguo Liang[1], Xinyue Mei[4], Yuanfa Feng[1], Zhaodong Han[2], Funeng Jiang[2], Shengbang Yang[2], Yongding Wu[2], Huijing Tan[2], Junchen Liu[5], Huichan He[1,*], Weide Zhong[1,2,3,4,*]

Affiliations

1. Guangdong Provincial Key Laboratory of Urology, the First Affiliated Hospital of Guangzhou Medical University, Guangzhou, 510230, China.
2. Guangdong Key Laboratory of Clinical Molecular Medicine and Diagnostics, The Second Affiliated Hospital, School of Medicine, South China University of Technology, Guangzhou, 510180, China.
3. Macau Institute for Applied Research in Medicine and Health, Macau University of Science and Technology, Macau, 999078, China.
4. Guangzhou National Laboratory, Guangzhou, Guangdong Province, 510320, China.
5. Department of Integrative Biology and Pharmacology, McGovern Medical School, University of Texas Health Science Center, Houston, Texas 77030, USA.

[*]**Corresponding author.** Weide Zhong. E-mail: zhongwd2009@live.cn

Co-Corresponding author. Shaoyou Liu. E-mail:doctorsyl@outlook.com. Huichan He. E-mail: xiaohejian@21cn.com.

[#]These authors contributed equally to this work.

Running title: Depletion of ACACA Facilitates EMT of PCa

Keywords: fatty acid biosynthesis, ACACA, MAPK, EMT, prostate cancer, metastasis

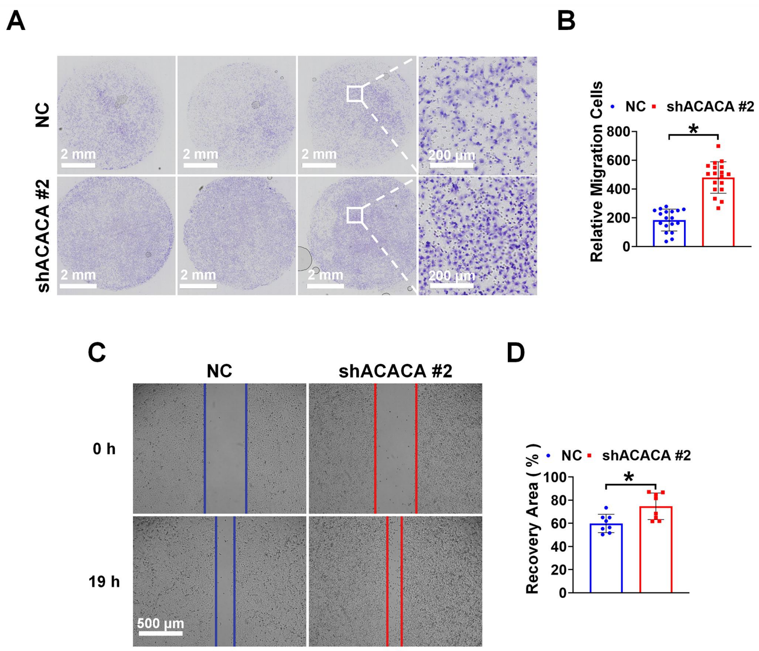

Figure S1. Validation of increased metastatic potential in PCa cells through the second shRNA to deplete ACACA.

(A-B) Assessment of the migration potential of ACACA-depleted DU145 cells using Transwell assay. **(C-D)** Assessment of the migration potential of ACACA-depleted DU145 cells using wound healing assay. Quantification of the relative data using ImageJ. *$p < 0.05$

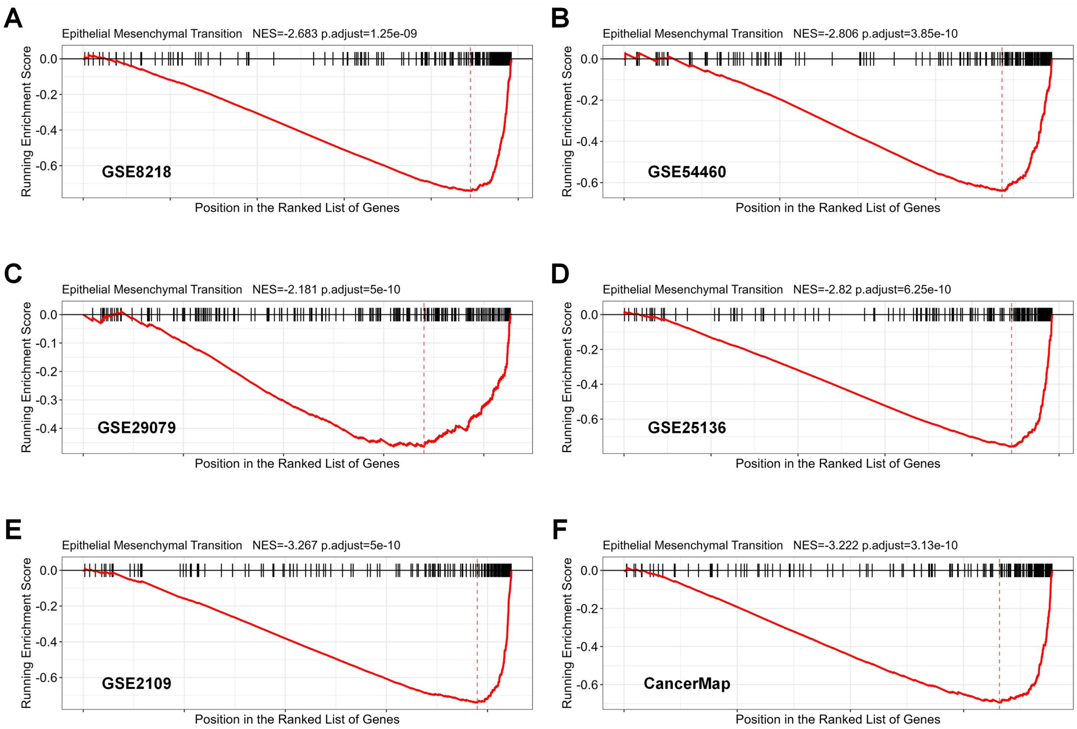

Figure S2. GSEA analysis of ACACA expression and EMT signaling pathways in specified datasets.

(A-F) GSEA analysis of ACACA expression (high vs low) and Epithelial-Mesenchymal Transition (EMT) signaling pathways in specified datasets (GSE8218, GSE54460, GSE29079, GSE25136, GSE2109, CancerMap), with adjustedp<0.05.

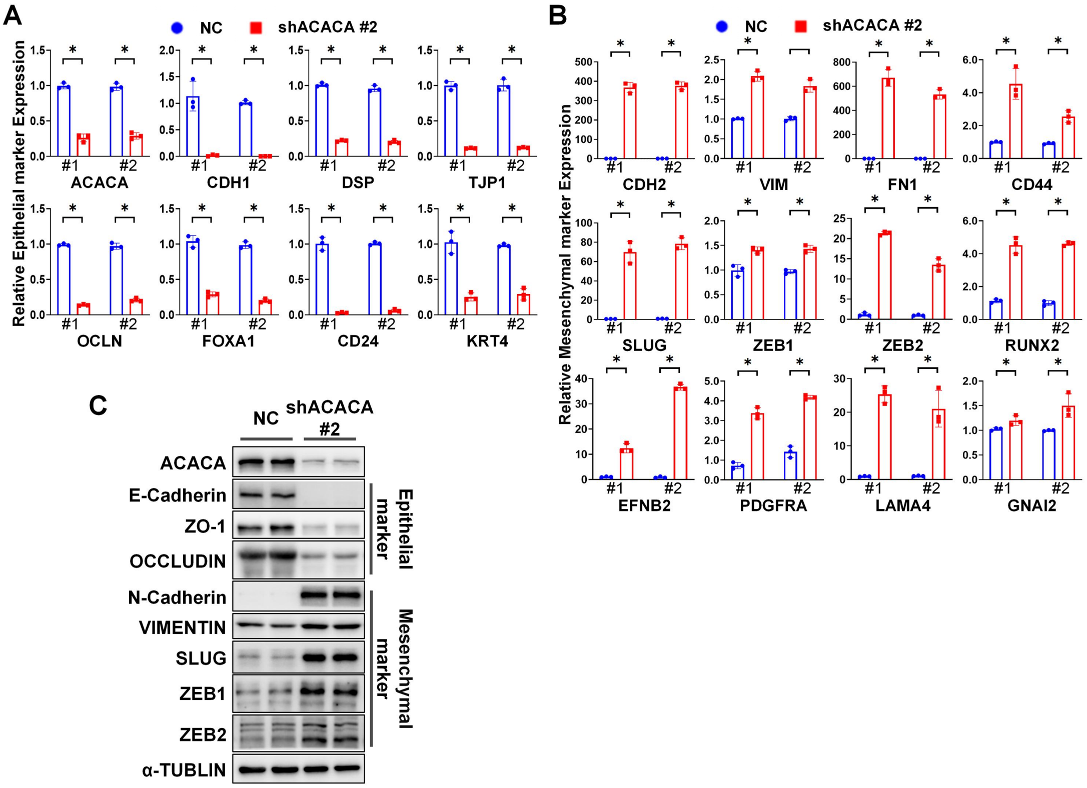

Figure S3. Validation of EMT induction in PCa cells using qPCR and Western blot through the second shRNA to deplete ACACA.

(A-B) Quantification of qPCR analysis results of EMT biomarker-related gene expression in ACACA-depleted DU145 cells, using two primers (#1, primer 1; #2, primer 2). **(C)** Western blot analysis of the specified protein levels in ACACA-depleted DU145 cells. *$p < 0.05$

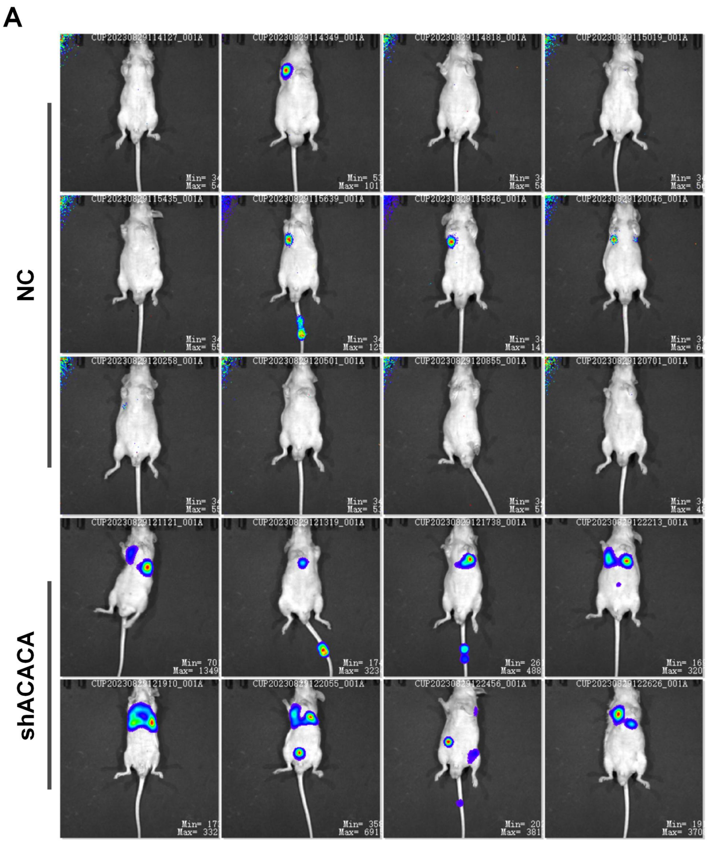

Figure S4. Live images of indicated luciferase-labeled mice were taken after 51 days.

(A) Indicated luciferase-labeled DU145 cells were administered to BALB/c nude mice via the tail vein, and live images were taken after 51 days.

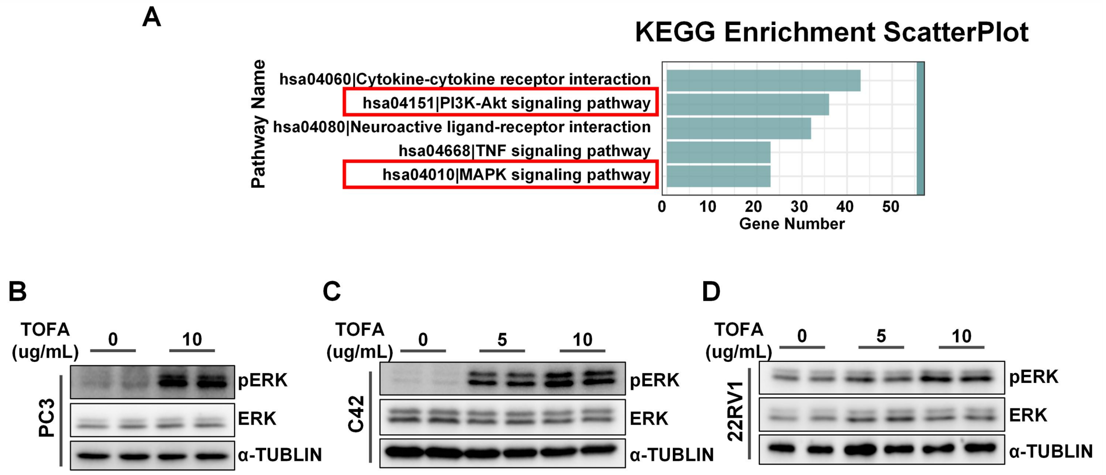

Figure S5. Depletion of ACACA activates the MAPK/ERK pathway

(A) Pathways significantly enriched in ACACA-depleted PC3 cells. **(B)** Western blot analysis of the specified protein levels in ACACA inhibitor (TOFA, 48 hours)-treated PC3, C42 and 22RV1 PCa cells.

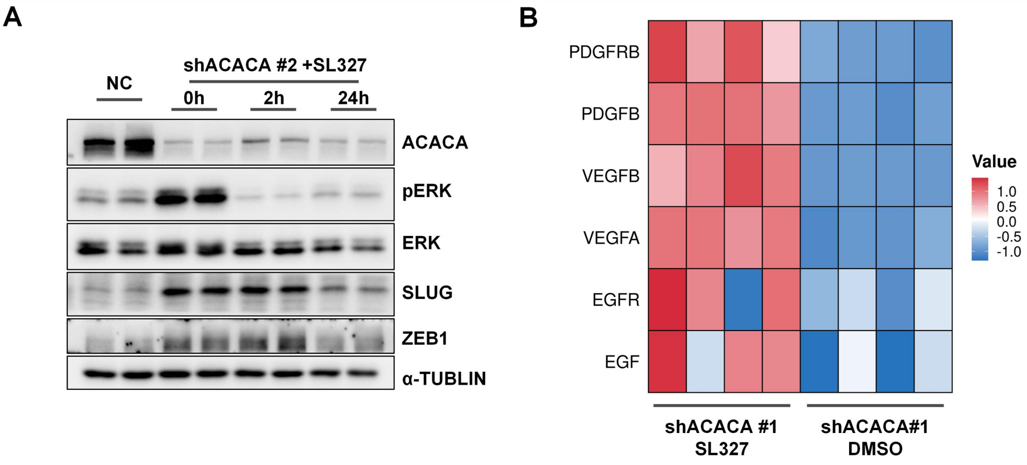

Figure S6. Inhibition of the MAPK pathway suppresses the expression of EMT transcription factors and exhibits negative feedback characteristics.

(A) Western blot analysis of the specified protein levels in ACACA-depleted DU145 cells treated with SL327 (10 μM) using the second shRNA sequece. (B) Heatmap based on RNA sequencing results of ACACA-depleted DU145 cells following SL327 treatment (10 μM, 24 hours).

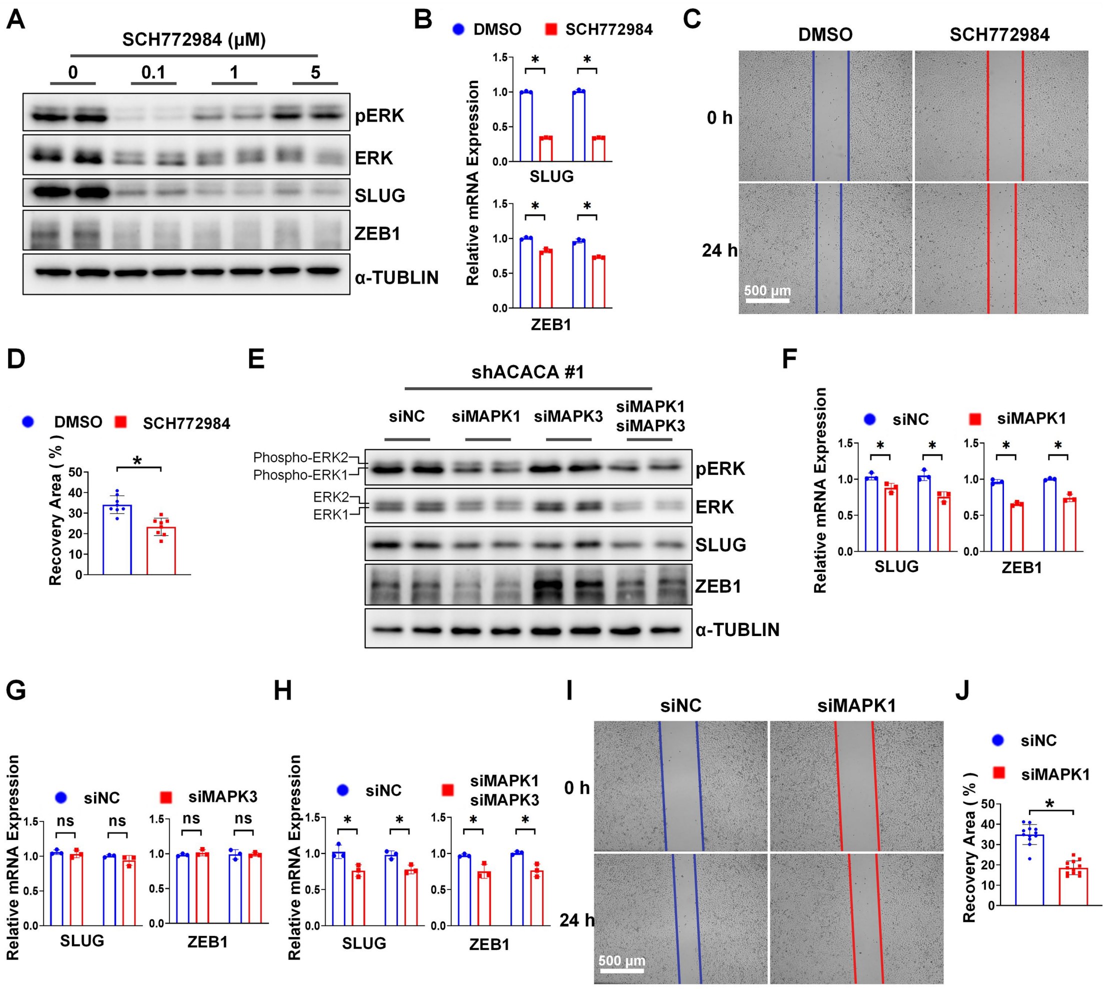

Figure S7. Inhibition of MAPK/ERK signaling attenuates migration of ACACA-depleted PCa cells.

(A) Western blot analysis of the specified protein levels in ACACA-depleted DU145 cells following treatment with an ERK inhibitor (SCH772984, 24 hours). **(B)** Quantification of qPCR analysis results of the specified gene expression in ACACA-depleted DU145 cells following treatment with an ERK inhibitor (SCH772984, 1 μM, 24 hours). **(C and D)** Assessment of the migration potential of ACACA-depleted DU145 cells following treatment with ERK inhibitors (SCH772984, 1 μM) using wound healing assays. **(E-H)** Western blot (E) and qPCR (F-H) analyses results of the specified protein levels and gene expression, respectively, in ACACA-depleted DU145 cells upon MAPK1 (also known as ERK2) and MAPK3 (also known as ERK1) silencing. **(I and J)** Assessment of the migration potential of ACACA-depleted DU145 cells following MAPK1 silencing using wound healing assays. Quantification of the indicated results using ImageJ. qPCR analysis results obtained using two different primers. *$p < 0.050$

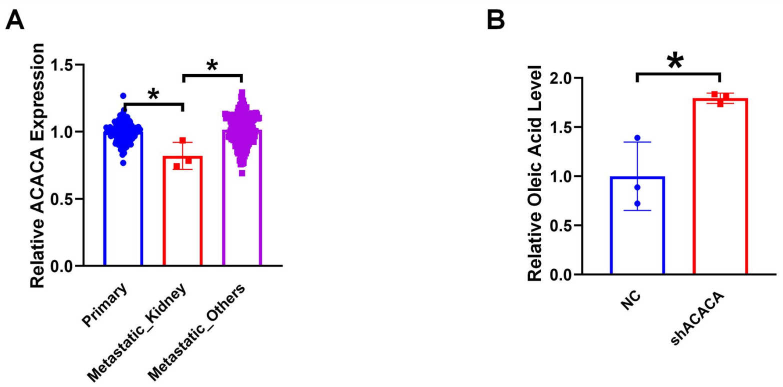

Figure S8. Expression of the ACACA gene in prostate cancer and oleic acid level in PCa cell after depletion of ACACA.

(A) Comparative analysis of ACACA expression in prostate cancer kidney metastases versus primary or metastases in other locations across three datasets (GSE6919-GPL93, GSE6919-GPL8300, GSE77930). **(B)** Oleic acid level after depletion of ACACA in DU145 cells.

Table S1. Sequences used for shRNA or siRNA.

gene	sense/antisense	sequence
shACACA #1	5'-3'	TACAAGGGATACAGGTATTTA
shACACA #2	5'-3'	CCAGCACTCTCGATTCATAAT
siMAPK1	sense (5'-3')	UACAGGUCAGUCUCCAUCAdTdT
	antisense (5'-3')	CCUGCUGGACCGGAUGWUAdTdT
siMAPK3	sense (5'-3')	GACUCCAAAGCUCUGGACUdTdT
	antisense (5'-3')	AGUCCAGAGCUUUGGAGUCdTdT
siSLUG	sense（5'-3'）	CAUAUUCGGACCCACACAUdTdT
	antisense（5'-3'）	AUGUGUGGGUCCGAAUAUGdTdT

Table S2. Antibodies involved in the study.

Primary Antibodies	Companies	Cat.#	Dilution
ACC1	Cell Signaling Technology	3676S	1:1000
Vimentin	Cell Signaling Technology	5741	1:1000
N-Cadherin	Cell Signaling Technology	13116	1:1000
Snail	Cell Signaling Technology	3879	1:1000
SLUG	Cell Signaling Technology	9585	1:1000
ZEB1	Cell Signaling Technology	3396	1:1000
OCCLUDIN	Cell Signaling Technology	91131	1:1000
Phospho-p44/42 MAPK (Erk1/2) (Thr202/Tyr204)	Cell Signaling Technology	4370	1:1000
p44/42 MAPK (Erk1/2)	Cell Signaling Technology	4695	1:1000
E-cadherin	Proteintech	20874-1-AP	1:1000
ZO-1	Proteintech	21773-1-AP	1:1000
ZEB2	Proteintech	14026-1-AP	1:1000
alpha Tubulin	abcam	ab176560	1:2000
Phospho-AKT1/2/3 (Ser473) Antibody	Affinity Bioscience	AF0016	1:1000
pan-AKT1/2/3 Antibody	Affinity Bioscience	AF6261	1:1000
beta Actin	Affinity Bioscience	AF7018	1:2000

Table S3. Primer sequences for qPCR.

Gene		Sequence (5' -> 3')	
		Forward Primer	Reverse Primer
ACTIN	#1	AGCGAGCATCCCCCAAAGTT	GGGCACGAAGGCTCATCATT
ACACA	#1	CATGCGGTCTATCCGTAGGTG	GTGTGACCATGACAACGAATCT
	#2	AGGAGCTGTCTATTCGGGGT	GGTCGCTCAGCCTGTACTTT
CDH1	#1	CGAGAGCTACACGTTCACGG	GGGTGTCGAGGGAAAAATAGG
	#2	ATTTTTCCCTCGACACCCGAT	TCCCAGGCGTAGACCAAGA
DSP	#1	GCAGGATGTACTATTCTCGGC	CCTGGATGGTGTTCTGGTTCT
	#2	CAGCCCTGTGATGCTTACCAG	ACTCTGACAAGTGTAGCCTCC
TJP1	#1	CAACATACAGTGACGCTTCACA	CACTATTGACGTTTCCCCACTC
	#2	CTGGTGAAATCCCGGAAAAATGA	TTGCTGCCAAACTATCTTGTGA
OCLN	#1	ACAAGCGGTTTTATCCAGAGTC	GTCATCCACAGGCGAAGTTAAT
	#2	GACTTCAGGCAGCCTCGTTAC	GCCAGTTGTGTAGTCTGTCTCA
FOXA1	#1	CTACTACGCAGACACGCAG	CCGCTCGTAGTCATGGTG
	#2	GCAATACTCGCCTTACGGCT	TACACACCTTGGTAGTACGCC
CD24	#1	CTCCTACCCACGCAGATTTATTC	AGAGTGAGACCACGAAGAGAC
	#2	GCGCGGACTTTTCTTTTGGG	TCACTGGAATAAATCTGCGTGG
KRT4	#1	CGCGAACAGATCAAGCTCCT	GGGGCTCAAGGTTTTTGCTG
	#2	CTCTTTGAGACCTACCTCAGTGT	GGCTGCTGTGCGTTTGTTG
CDH2	#1	TCAGGCGTCTGTAGAGGCTT	ATGCACATCCTTCGATAAGACTG
	#2	AGCCAACCTTAACTGAGGAGT	GGCAAGTTGATTGGAGGGATG
VIM	#1	GACGCCATCAACACCGAGTT	CTTTGTCGTTGGTTAGCTGGT
	#2	AGTCCACTGAGTACCGGAGAC	CATTTCACGCATCTGGCGTTC
FN1	#1	CGGTGGCTGTCAGTCAAAG	AAACCTCGGCTTCCTCCATAA
	#2	GAGAATAAGCTGTACCATCGCAA	CGACCACATAGGAAGTCCCAG
CD44	#1	CTGCCGCTTTGCAGGTGTA	CATTGTGGGCAAGGTGCTATT
	#2	AGGGATATCGCCAAACACCC	ATGGCTGGTATGAGCTGAGG
SLUG	#1	CGAACTGGACACACATACAGTG	CTGAGGATCTCTGGTTGTGGT
	#2	TGTGACAAGGAATATGTGAGCC	TGAGCCCTCAGATTTGACCTG
ZEB1	#1	GATGATGAATGCGAGTCAGATGC	ACAGCAGTGTCTTGTTGTTGT
	#2	CAGCTTGATACCTGTGAATGGG	TATCTGTGGTCGTGTGGGACT
ZEB2	#1	CAAGAGGCGCAAACAAGCC	GGTTGGCAATACCGTCATCC
	#2	GCGATGGTCATGCAGTCAG	CAGGTGGCAGGTCATTTTCTT
RUNX2	#1	TGGTTACTGTCATGGCGGGTA	TCTCAGATCGTTGAACCTTGCTA
	#2	TCAACGATCTGAGATTTGTGGG	GGGGAGGATTTGTGAAGACGG
EFNB2	#1	ACTGTGCCAAACCAGACCA	ACCCCTCCCTCCTGGTTAT
	#2	TATGCAGAACTGCGATTTCCAA	TGGGTATAGTACCAGTCCTTGTC
PDGFRA	#1	TGGCAGTACCCCATGTCTGAA	CCAAGACCGTCACAAAAAGGC

	#2	TTTTTGTGACGGTCTTGGAAGT	TGTCTGAGTGTGGTTGTAATAGC
LAMA4	#1	CCACACTCGTCCTTCTCTCTC	AGTTTCCGAACTGACCTAGCC
	#2	AGGATACTGTGTGACTACTGACG	TGAACGATAGGGTAGAAGCTGAA
GNAI2	#1	AAGTGACTCCGTGCCTTG	GAACAGCCCTTGGAACC
	#2	TACCGGGCGGTTGTCTACA	GGGTCGGCAAAGTCGATCTG
